# Clinical Outcomes of Sequential Distalization with Clear Aligners: A Systematic Review and Meta-Analysis

**DOI:** 10.3390/dj14090611

**Published:** 2026-09-20

**Authors:** Martin Baxmann, Márton Zsoldos, Krisztina Kárpáti

**Affiliations:** Department of Orthodontics and Paediatric Dentistry, Faculty of Dentistry, University of Szeged, Tisza Lajos körút 64–66, H-6720 Szeged, Hungarykarpati.krisztina.margit@szte.hu (K.K.)

**Keywords:** clear aligners, sequential distalization, molar distalization, orthodontics, temporary anchorage devices, anchorage preservation

## Abstract

**Background/Objectives**: Sequential distalization with clear aligners is increasingly used as a non-extraction approach for correcting Class II and selected Class III malocclusions. However, evidence remains heterogeneous regarding treatment protocols, anchorage strategies, and clinical outcomes. This systematic review and meta-analysis evaluated the clinical outcomes of sequential distalization with clear aligners, including achieved posterior tooth movement, predictability, biomechanical effects, and other clinically relevant outcomes. **Methods**: This systematic review was conducted according to PRISMA 2020 guidelines, and the protocol was prospectively registered in the Open Science Framework. PubMed, Embase, Scopus, Web of Science Core Collection, and the Cochrane Library were searched from inception through 1 July 2026. Eligible clinical studies evaluating sequential distalization with clear aligners were included. Random-effects meta-analyses were performed for sufficiently comparable outcomes, and certainty of evidence was assessed using GRADE. **Results**: Twenty-eight studies met the inclusion criteria. Pooled mean achieved distalization was 1.72 mm (95% CI, 1.02–2.42) for the maxillary first molar and 1.76 mm (95% CI, 1.12–2.40) for the maxillary second molar. Pooled distal tipping was 5.33° (95% CI, 1.37–9.30) for first molars and 5.77° (95% CI, −6.84–18.38) for second molars. First-molar intrusion was 0.58 mm (95% CI, 0.05–1.10). Movement predictability varied substantially across studies. Certainty of evidence was very low across clinically important domains. **Conclusions**: Sequential distalization with clear aligners may achieve moderate maxillary molar distalization but is accompanied by variable movement expression and biomechanical effects. Heterogeneous protocols and limited study quality restrict the certainty and generalizability of current estimates.

## 1. Introduction

Clear aligner therapy has transformed contemporary orthodontic practice by providing an esthetic, comfortable, and digitally planned alternative to conventional fixed appliances [1,2]. Initially indicated primarily for relatively simple malocclusions, advances in orthodontic materials, computerized treatment planning, optimized attachment systems, and adjunctive biomechanics have substantially expanded the range of cases that can be managed with removable aligners [2,3]. Among these advances, sequential distalization has emerged as an important non-extraction strategy for correcting Class II malocclusions and selected Class III malocclusions while preserving facial esthetics and avoiding premolar extractions [4,5,6].

Sequential distalization involves the staged posterior movement of teeth, typically beginning with the second molars and progressing anteriorly until the desired occlusal relationship is achieved [4]. Digital treatment planning enables clinicians to prescribe individualized tooth movements while incorporating adjunctive mechanics such as optimized attachments, intermaxillary elastics, power arms, and temporary anchorage devices (TADs) to improve movement predictability, reinforce anchorage, and minimize undesirable reciprocal tooth movement [7]. Despite these technological advances, achieving predictable distalization remains biomechanically challenging. Clinical investigations have reported considerable variability in the amount of posterior movement achieved, the predictability of planned tooth movement, and the occurrence of unintended effects, including anterior anchorage loss, molar tipping, vertical tooth movement, root resorption, and periodontal changes [8,9,10]. Contemporary studies have also expanded the range of evaluated outcomes through the use of cone-beam computed tomography (CBCT), digital model superimposition, and other three-dimensional assessment methods [11,12].

Recent systematic reviews have evaluated maxillary and mandibular distalization, movement predictability, vertical control, periodontal effects, root resorption, treatment efficiency, and different anchorage strategies [4,13,14,15]. Previous quantitative syntheses have focused specifically on molar distalization and associated sagittal effects [4] or on dental and skeletal vertical changes [14], with both reviews excluding TAD-assisted distalization. Other reviews have examined movement predictability [13] and the use of temporary anchorage devices in clear-aligner therapy [15]. Although the present review includes primary studies represented in these earlier syntheses, it incorporates an updated evidence base through July 2026 and a broader range of treatment protocols and outcomes, including TAD-assisted distalization, maxillary and mandibular distalization, movement predictability, anchorage effects, three-dimensional biomechanical changes, biological outcomes, treatment duration and refinement, stability, adverse effects, and patient-reported outcomes within a single review. Therefore, the objective of this systematic review and meta-analysis is to evaluate the clinical outcomes of sequential distalization using clear aligners by synthesizing evidence regarding the magnitude of achieved posterior tooth distalization, anchorage preservation, treatment predictability, vertical changes, treatment duration, adverse effects, and other clinically relevant treatment outcomes.

## 2. Materials and Methods

This systematic review was conducted and reported in accordance with the Preferred Reporting Items for Systematic Reviews and Meta-Analyses (PRISMA 2020) statement (Supplementary Table S5). The review protocol was prospectively registered in the Open Science Framework (OSF) (Registration: https://osf.io/7b894) (accessed on 30 July 2026). OSF was used to provide a publicly accessible, time-stamped prospective record of the review protocol. Minor administrative amendments made before study commencement were documented within the registration record.

Eligibility criteria were established a priori using the Population, Intervention, Comparator, Outcomes, and Study Design (PICOS) framework (Table 1). Studies were eligible if they evaluated sequential distalization using commercially available clear aligner systems in human participants and reported one or more predefined clinical outcomes. Randomized controlled trials, prospective and retrospective cohort studies, comparative clinical studies, and observational clinical studies were eligible for inclusion. Studies involving finite element analyses, animal, cadaveric, or in vitro models, case reports, case series, conference abstracts, editorials, narrative reviews, systematic reviews, meta-analyses, and studies not evaluating sequential distalization with clear aligners or not reporting clinically relevant outcomes were excluded.

Electronic searches were conducted in PubMed (MEDLINE), Embase, Scopus, Web of Science Core Collection, and the Cochrane Library from database inception through 1 July 2026. Additional studies were identified through manual screening of reference lists of included studies and relevant systematic reviews, together with forward citation tracking using Google Scholar, Scopus, and Web of Science. Database-specific search strategies combined controlled vocabulary and free-text terms related to clear aligners and sequential distalization. Equivalent search strategies were adapted for each database while maintaining the same conceptual framework. The complete search strategies are provided in Supplementary File S1. No language restrictions were applied to the electronic searches. Trial registries and other dedicated grey-literature sources were not searched.

All retrieved records were imported into Zotero (Corporation for Digital Scholarship, Vienna, VA, USA) for duplicate removal and subsequently uploaded into Covidence (Veritas Health Innovation, Melbourne, Australia) for study screening. Two reviewers independently screened titles and abstracts according to the predefined eligibility criteria, followed by full-text assessment of potentially eligible studies. Disagreements were resolved through discussion and, when necessary, consultation with a third reviewer. Reasons for exclusion during full-text screening were documented, and the study selection process was summarized using a PRISMA 2020 flow diagram.

A standardized data extraction form was pilot-tested before full data extraction. Two reviewers independently extracted study characteristics, participant demographics, intervention and comparator characteristics, aligner systems, distalization protocols, adjunctive biomechanics, outcome measures, measurement methods, and follow-up information. Quantitative data were extracted for distalization amount, movement predictability, anchorage preservation, vertical changes, treatment duration, biological outcomes, treatment stability, adverse events, and patient-reported outcomes when available. Disagreements were resolved through discussion. The complete data extraction form is provided in Supplementary File S2.

Risk of bias was independently assessed by two reviewers using study design-specific appraisal tools. Randomized controlled trials were evaluated using the Cochrane Risk of Bias 2 (RoB 2) tool. Nonrandomized comparative studies were assessed using the Risk Of Bias In Non-randomized Studies of Interventions (ROBINS-I) tool, whereas uncontrolled clinical studies were evaluated using the Joanna Briggs Institute (JBI) Critical Appraisal Checklist for Quasi-Experimental Studies. This tool was selected for uncontrolled studies in which the clinical intervention preceded outcome assessment and treatment-related changes were evaluated within a single group, including pre- and post-intervention designs, regardless of whether data collection was prospectively or retrospectively. For studies assessed using the JBI checklist, individual appraisal items were evaluated without assigning an overall categorical risk-of-bias judgment because the instrument does not specify thresholds for deriving global ratings. Disagreements were resolved through discussion or consultation with a third reviewer. Risk-of-bias assessments were summarized in tabular form.

Study characteristics and findings were summarized descriptively. Clinical and methodological heterogeneity were evaluated by examining differences in study design, participant characteristics, aligner systems, distalization protocols, adjunctive biomechanics, outcome definitions, and measurement methods. Compatibility of outcome measures was assessed before quantitative synthesis. Studies reporting different anatomical landmarks, incompatible measurement frameworks, or insufficient statistical information were synthesized narratively rather than pooled.

Quantitative synthesis was performed only when at least two studies reported sufficiently comparable interventions, outcome definitions, and quantitative data. Continuous outcomes were synthesized as pooled single-group means using random-effects models. Between-study variance (τ^2^) was estimated using restricted maximum likelihood (REML), with Hartung–Knapp t-based 95% confidence intervals. Sampling variances were calculated as SD^2^/n using reported or reconstructed study-level standard deviations and the corresponding analytic sample sizes. When a standard deviation was not directly reported, it was reconstructed from the available confidence interval when sufficient information was provided. Statistical heterogeneity was assessed using Cochran’s Q test, the I^2^ statistic, and τ^2^. Prediction intervals were calculated for analyses containing at least three studies.

When a study reported multiple eligible treatment groups contributing to the same synthesis, relevant groups were combined before pooling to avoid double counting. Sensitivity analyses were conducted where appropriate to evaluate the influence of analytical unit, measurement framework, reconstructed variance estimates, and potentially influential studies. Formal subgroup analyses according to clinical factors such as treatment stage, measurement method, distalization protocol, and skeletal anchorage were considered but were not performed because the small number of studies within relevant strata and heterogeneity in outcome reporting did not support reliable subgroup estimates. Because some studies reported tooth-level rather than participant-level estimates without adjustment for within-participant clustering, participant-level sensitivity analyses were performed when sufficient studies were available. Outcomes that could not be quantitatively synthesized because of methodological or clinical heterogeneity were summarized narratively in accordance with the Synthesis Without Meta-analysis (SWiM) reporting guideline. Statistical analyses were performed using R version 4.4.2 (R Foundation for Statistical Computing, Vienna, Austria).

Publication bias was explored visually using funnel plots for the primary meta-analyses of maxillary first- and second-molar distalization. Because fewer than ten studies contributed to each primary meta-analysis, funnel plots were interpreted descriptively and formal statistical tests for funnel plot asymmetry (e.g., Egger’s regression test) were not performed, consistent with current methodological recommendations. The certainty of evidence for clinically important outcomes was assessed using the Grading of Recommendations Assessment, Development and Evaluation (GRADE) framework. Certainty was evaluated across the domains of risk of bias, inconsistency, indirectness, imprecision, and publication bias and was rated as high, moderate, low, or very low. GRADE assessments were conducted separately for each outcome.

## 3. Results

### 3.1. Study Selection

**Figure 1:**
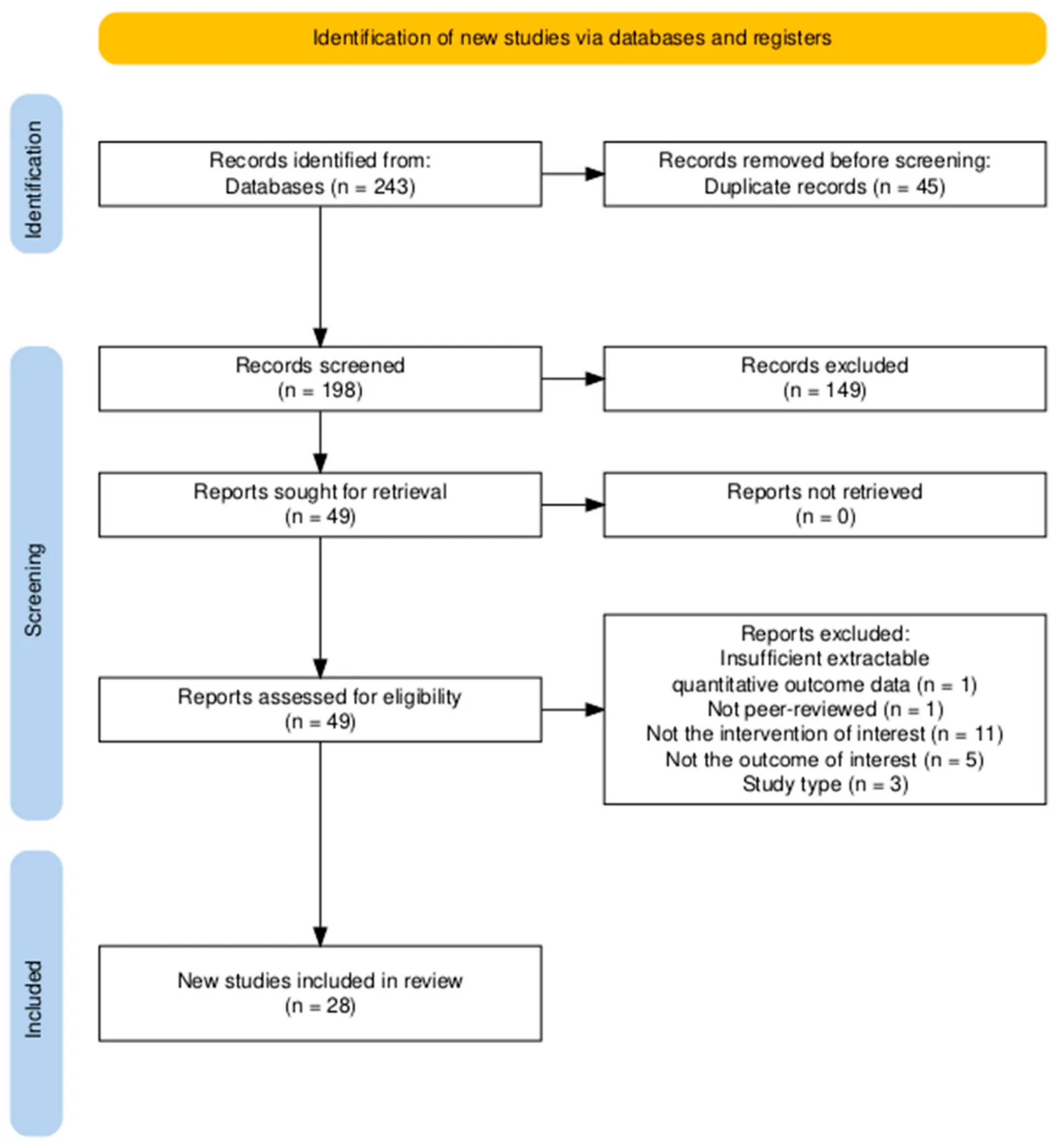
PRISMA flow diagram.

The database search identified 243 records. After the removal of 45 duplicate records, 198 unique records remained for title and abstract screening. Following screening, 149 records were excluded, and the full texts of the remaining 49 studies were assessed for eligibility. All full-text reports were successfully retrieved. Twenty-one studies were excluded after full-text review for the following reasons: not the intervention of interest (*n* = 11), not the outcome of interest (*n* = 5), study type (*n* = 3), not peer-reviewed (*n* = 1), and insufficient extractable quantitative outcome data (*n* = 1) (Supplementary Table S1). Ultimately, 28 studies met the eligibility criteria and were included in the qualitative synthesis (Figure 1).

### 3.2. Study Characteristics

The characteristics of the included studies are summarized in Supplementary Table S2. Twenty-eight studies published between 2014 and 2026 met the inclusion criteria, with most published within the past five years. Studies were conducted predominantly in China and Italy, with additional investigations from Germany, Canada, Egypt, the United States, Türkiye, Russia, Thailand, Japan, and multicenter collaborations. Retrospective clinical studies comprised the largest proportion of the evidence base, alongside prospective clinical studies, randomized controlled trials, comparative cohort studies, and planned-versus-achieved analyses.

Sample sizes ranged from seven to 46 participants, although several studies reported tooth- or root-level analyses. Most investigations enrolled adolescents or adults with Class II malocclusions undergoing non-extraction treatment, while relatively few evaluated Class II subdivision malocclusions, selected Class III cases, or mandibular crowding. Invisalign was the most frequently investigated aligner system, and the majority of studies focused on maxillary sequential distalization, with comparatively few evaluating mandibular distalization or combined maxillary and mandibular protocols. Outcome assessment most commonly relied on CBCT, three-dimensional digital model superimposition, or lateral cephalometry, with assessment time points varying from post-distalization evaluations to post-treatment evaluations and planned-versus-achieved comparisons.

### 3.3. Characteristics of the Distalization Protocols

Sequential distalization protocols generally followed a posterior-to-anterior sequence, beginning with the second molars and progressing to the first molars before movement of the premolars and anterior teeth until the desired molar relationship was achieved [16,17,18,19,20,21,22,23]. Approximately 50% overlapping tooth movement between adjacent teeth was the most frequently reported staging strategy, although 33% sequencing, V-pattern mandibular distalization, unilateral distalization, and total-arch distalization were also described [20,21,22,24,25,26,27,28,29]. Isolated maxillary molar distalization without simultaneous anterior tooth movement was also reported [30]. Class II elastics were the most frequently reported auxiliary anchorage strategy during maxillary distalization, whereas skeletal anchorage, using miniscrews, micro-implants, infrazygomatic crest temporary anchorage devices, or no standardized auxiliary anchorage, was incorporated into other treatment protocols [16,17,18,20,22,23,24,31,32,33,34,35,36,37]. Comparative protocols included skeletal anchorage relative to Class II elastics and clear aligners relative to fixed appliances using similar distalization strategies [24,25,38,39]. Sequential distalization without standardized auxiliary anchorage was also described [26,28,29,30]. When reported, programmed tooth movement was typically 0.20–0.25 mm per aligner, aligners were replaced every 7–10 days, and recommended wear time was at least 22 h per day [16,20,23,24,25,28,29,30]. Posterior attachments, including rectangular vertical attachments, were incorporated into most treatment protocols, although attachment design and placement were frequently individualized according to the digital treatment plan [16,17,18,21,22,24,25,26,31,32,33,34,35,39,40,41]. Patient compliance, refinement protocols, and the number of refinement aligners were reported when available [25,34,35].

### 3.4. Magnitude of Achieved Distalization

The pooled random-effects estimate for maxillary first-molar distalization was 1.72 mm (95% CI, 1.02–2.42 mm), whereas the pooled estimate for maxillary second-molar distalization was 1.76 mm (95% CI, 1.12–2.40 mm) (Table 2). Heterogeneity was substantial for both analyses, with I^2^ values of 96.5% and 86.5%, respectively. The corresponding 95% prediction intervals ranged from −0.28 to 3.72 mm for the maxillary first molar and from 0.04 to 3.48 mm for the maxillary second molar. Individual studies reported maxillary first-molar distalization ranging from approximately 1.25 to 2.63 mm and second-molar distalization ranging from 1.41 to 2.42 mm. Across the included studies, distalization of the second molar generally equaled or exceeded that of the first molar [19,22,23,26,40]. In sensitivity analyses restricted to participant-level estimates, pooled distalization was 2.07 mm (95% CI, 1.04–3.10 mm) for the maxillary first molar and 1.96 mm (95% CI, 0.64–3.28 mm) for the maxillary second molar. The pooled random-effects estimate for mandibular first-molar distalization was 1.87 mm (95% CI, −0.32–4.06 mm), whereas the pooled estimate for mandibular second-molar distalization was 1.96 mm (95% CI, −2.06–5.97 mm) (Table 2). Heterogeneity was 79.7% for the first-molar analysis and 37.9% for the second-molar analysis. Individual studies reported mandibular first-molar distalization ranging from 0.85 to 2.68 mm and second-molar distalization ranging from 1.44 to 2.15 mm [25,28,29]. Visual inspection of funnel plots for the primary meta-analyses revealed no visually apparent asymmetry; however, interpretation was limited by the small number of included studies (Supplementary Figure S1).

### 3.5. Predictability of Tooth Movement

Reported accuracy of maxillary molar distalization ranged from 19.21% to 88.4% across the included studies [18,21,23,27,30,34,35,41]. Reported accuracy was highest (86.9–88.4%) for isolated maxillary molar distalization, whereas accuracy following the distalization phase of comprehensive treatment was 75.70% for the maxillary first molar and 75.01% for the second molar [23,30]. Reported movement expression for planned-versus-achieved distalization ranged from approximately 31.00% to 40.11% for the maxillary first molar and from 31.06% to 35.39% for the second molar [21,35,41]. Predictability was evaluated using several outcome definitions, including percentage accuracy, planned-versus-achieved displacement, movement expression, derotation, and prediction error [18,21,23,27,30,34,35,41]. Reduced movement expression with increasing programmed distalization and incomplete expression of unilateral distalization were also reported [27,34]. Differences between planned and achieved derotation were reported in analyses of rotational tooth movement [18].

### 3.6. Biomechanical Effects

Maxillary first-molar distal tipping demonstrated a pooled random-effects estimate of 5.33° (95% CI, 1.37–9.30°), whereas the pooled estimate for maxillary second-molar distal tipping was 5.77° (95% CI, −6.84° to 18.38°) (Table 2). Reported first-molar distal tipping ranged from 2.29° to 8.45°, whereas second-molar tipping ranged from 1.15° to 11.35° [19,25,26,40]. Maxillary first-molar intrusion demonstrated a pooled estimate of 0.58 mm (95% CI, 0.05–1.10 mm), whereas the pooled estimate for maxillary second-molar intrusion was 0.67 mm (95% CI, −1.14 to 2.48 mm) (Table 2). Reported first-molar intrusion ranged from 0.47 to 0.86 mm, whereas second-molar intrusion ranged from 0.58 to 0.89 mm [24,25,40]. Anterior anchorage changes included maxillary incisor proclination, linear incisor protrusion, overjet increase, mandibular incisor displacement, canine mesialization, and posterior molar relapse [20,23,24,25,26,28,29]. Reported torque outcomes included buccal crown torque and crown angulation, whereas reported rotational outcomes included distopalatal rotation, derotation, and planned-versus-achieved rotational discrepancies [18,19,21,28,40,41,42].

### 3.7. Biological and Secondary Treatment Effects

Reported external apical root resorption following maxillary molar distalization ranged from 0.43 ± 0.69 mm for the mesiobuccal root to 0.82 ± 0.87 mm for the palatal root [43]. Reductions in root length and changes in alveolar bone thickness were also reported, including palatal bone deposition adjacent to the maxillary first molars and reductions in buccal alveolar bone surrounding the first and second molars [33]. Temporomandibular joint outcomes included changes in condylar position, joint-space dimensions, and condylar morphology, with minimal post-treatment changes reported [31,32,35]. Comprehensive treatment duration ranged from approximately 33 to 34 months, whereas distalization phases ranged from approximately 4 to 11 months [16,25,26,29]. Refinement aligners were frequently required, with one study reporting a mean of 20 ± 12 additional aligners and another reporting that all treated patients required refinement [25,35]. Mesial movement of previously distalized molars during subsequent treatment stages was reported in one longitudinal study [23]. Soft-tissue changes were reported primarily following mandibular distalization [25,29,37]. Reported adverse effects included distal tipping, anchorage loss, vertical tooth movement, and root resorption [23,28,30,43].

### 3.8. Risk of Bias

Risk-of-bias assessments are summarized in Supplementary Table S3. Three randomized controlled trials were judged as having some concerns, with no studies classified as high risk of bias. Three nonrandomized comparative studies were judged as having serious risk of bias. The remaining 22 studies generally met JBI criteria for clear temporal relationships, consistent and reliable outcome measurement, and appropriate statistical analysis. However, none included a concurrent control group, and most relied on pre- and post-intervention measurements; follow-up limitations were identified in a small number of studies.

### 3.9. Certainty of Evidence

The certainty of evidence was rated as very low across the clinically important outcome domains assessed using GRADE. Downgrading was primarily attributable to risk of bias, substantial clinical and methodological inconsistency, and imprecision for several outcomes. Indirectness was also a concern for outcomes characterized by heterogeneous definitions, treatment protocols, or measurement approaches. Detailed GRADE judgments and reasons for downgrading are presented in Supplementary Table S4.

## 4. Discussion

Sequential distalization with clear aligners was associated with moderate maxillary molar distalization. Quantitative synthesis showed comparable distal movement of the maxillary first and second molars, whereas narrative evidence indicated that distalization may be accompanied by biomechanical changes, including distal tipping and slight molar intrusion. However, movement predictability varied considerably across studies, and quantitative synthesis was not feasible for several secondary outcomes because of substantial methodological heterogeneity. Collectively, these findings suggest that sequential distalization may achieve clinically relevant posterior tooth movement while demonstrating substantial variability in treatment expression and outcome reporting.

The pooled estimates identified in this review are generally consistent with previous systematic reviews reporting approximately 2 mm of maxillary molar distalization with clear aligners [4]. However, these pooled estimates should not be interpreted as a uniform expected amount of distalization because the contributing studies differed in patient selection, distalization protocols, adjunctive mechanics, and outcome assessment methods [18,23,25,26,36,40]. The broad prediction intervals, particularly the U6 interval extending below zero, further indicate that the pooled estimates represent average distal displacement across heterogeneous studies rather than a predictable treatment result in individual patients or clinical settings. Substantial heterogeneity was observed across the pooled analyses, which may reflect differences in staging protocols, adjunctive mechanics, attachment design, skeletal anchorage, imaging modality, and timing of outcome assessment [18,23,25,26,36,40]. These clinical and methodological differences are plausible contributors to the observed between-study variability, although their individual contributions could not be reliably estimated from the available evidence. Studies evaluating tooth movement immediately after completion of the distalization phase generally reported greater distalization and higher movement expression than investigations assessing outcomes after comprehensive treatment, emphasizing the importance of considering treatment stage when interpreting reported distalization outcomes [18,23,30,35]. The present review extends previous syntheses by integrating evidence relating to movement predictability, biomechanical effects, biological outcomes, treatment burden, and mandibular distalization within a single comprehensive review [4,13,14,15].

Reported movement predictability varied substantially across the included studies, with the highest accuracy observed when distalization was evaluated immediately after completion of the posterior distalization phase and lower accuracy reported when assessed after comprehensive treatment [18,21,23,30,35,41]. This difference appears to reflect differences in treatment stage and outcome assessment, which may contribute to the observed variation in predictability [18,23,30,35]. Several investigations demonstrated mesial movement of previously distalized molars during anterior alignment and space closure, indicating that a proportion of the space gained during distalization was subsequently utilized during comprehensive treatment [20,23,40].

Comparative studies suggested that clear aligners may provide greater control of molar tipping and vertical changes than fixed appliances during distalization, whereas skeletal anchorage was associated with greater bodily tooth movement without completely eliminating distal tipping [25,36,39]. Distal tipping nevertheless remained a common biomechanical finding, although its magnitude varied across studies [19,22,25,26,39,40]. This distinction is clinically relevant because distal crown displacement does not necessarily represent equivalent bodily tooth movement; accompanying distal tipping indicates that crown and root displacement may differ [28,39,41]. Accordingly, the pooled millimeter estimates should be interpreted specifically as maxillary molar displacement rather than as evidence of equivalent bodily distalization of the entire maxillary arch. Similarly, the occurrence of incisor proclination, anterior displacement, and canine mesialization indicates that sequential staging does not eliminate reciprocal anchorage effects, and anchorage control remains dependent on the distalization protocol and adjunctive mechanics [20,23,26]. A small amount of molar intrusion was also reported across several studies and may contribute to improved vertical control during posterior tooth movement [16,17,40]. Small amounts of external apical root resorption and localized alveolar bone remodeling were identified in CBCT-based studies, whereas temporomandibular joint assessments demonstrated minimal changes in condylar position and joint morphology following treatment [31,32,33,43]. However, the very low certainty of evidence, limited number of contributing studies, and the absence of long-term follow-up preclude definitive conclusions regarding the biological safety and long-term stability of sequential distalization.

Comprehensive treatment generally required approximately three years, with the distalization phase accounting for only a portion of the overall treatment [16,25,26,29]. Refinement aligners were frequently required, consistent with incomplete movement expression during comprehensive treatment [23,25,35]. Limited evidence also suggested that some loss of distalization occurred during subsequent treatment stages, indicating that posterior tooth movement continued to evolve throughout comprehensive treatment [23].

The available evidence suggests that sequential distalization with clear aligners may be a treatment option for achieving moderate maxillary molar distalization. However, these findings should be interpreted cautiously because the available evidence was dominated by observational studies, with relatively few randomized or comparative investigations. Risk-of-bias assessment further demonstrated that the randomized trials were judged to have some concerns, whereas all nonrandomized comparative studies were at serious risk of bias, primarily because of confounding. In addition, differences in treatment protocols, adjunctive mechanics, imaging modalities, measurement landmarks, and outcome definitions limited direct comparison across studies and precluded quantitative synthesis for several clinically relevant outcomes, including movement predictability, anchorage loss, torque, rotation, and long-term stability.

This review has several limitations that should be considered when interpreting the findings. The certainty of evidence was rated as very low across the clinically important outcome domains, reflecting concerns related primarily to risk of bias, inconsistency, and imprecision. Although quantitative synthesis was possible for several primary outcomes, the number of studies contributing to individual meta-analyses was small, and substantial heterogeneity in treatment protocols, treatment stage, measurement methods, and outcome definitions limit the generalizability of the pooled estimates. Accordingly, these estimates represent average values across heterogeneous studies and should not be interpreted as universally expected treatment effects across different aligner systems or clinical protocols. Many clinically relevant endpoints required narrative synthesis because of methodological and clinical heterogeneity. In addition, variance estimates were reconstructed for some studies when standard deviations were not directly reported, which introduces additional uncertainty into the quantitative synthesis. Several studies contributing quantitative data reported tooth-level outcomes without accounting for clustering within participants, which could underestimate sampling variance. Participant-level sensitivity analyses produced estimates of comparable magnitude, although with wider confidence intervals, and did not materially alter the overall interpretation of the primary maxillary findings. Future research should prioritize well-designed prospective comparative studies with standardized treatment protocols, outcome definitions, and measurement methods to improve comparability across investigations. Longer-term follow-up is also needed to clarify treatment stability, biological outcomes, and patient-reported outcomes following sequential distalization.

## 5. Conclusions

Sequential distalization with clear aligners may achieve moderate maxillary molar distalization. Distalization may be accompanied by biomechanical changes, including distal tipping and slight molar intrusion, whereas movement predictability varies according to treatment stage and assessment methodology. Although these findings support the potential for clinically relevant maxillary molar distalization with clear aligners, variability in treatment protocols, outcome reporting, and study quality limits direct comparison across studies. Further well-designed prospective comparative studies using standardized outcome measures are needed to strengthen the evidence base and improve the consistency of future investigations.

## Figures and Tables

**Table 1:** PICOS Framework.

Component	Eligibility Criteria
Population	Adolescents or adults undergoing sequential distalization with clear aligners for Class II or selected Class III malocclusions
Intervention	Sequential distalization using any commercially available clear aligner system, with or without adjunctive biomechanics (e.g., temporary anchorage devices, Class II elastics, attachments)
Comparator	Alternative distalization protocols, different anchorage strategies, different aligner systems, fixed appliances, or no comparator
Primary Outcome	Amount of posterior tooth distalization
Secondary Outcomes	Anchorage preservation, movement predictability, vertical changes, treatment duration, root resorption, periodontal outcomes, treatment stability, adverse events, and patient-reported outcomes
Study Designs	Randomized controlled trials, prospective and retrospective cohort studies, comparative clinical studies, and observational clinical studies

**Table 2:** Summary of quantitative syntheses of sequential distalization outcomes.

Outcome	Studies (*n*)	Pooled Estimate (95% CI) ^1^	I^2^ (τ^2^)
Maxillary first-molar distalization	7	1.72 mm (1.02–2.42)	I^2^ = 96.5%; τ^2^ = 0.526
Maxillary second-molar distalization	6	1.76 mm (1.12–2.40)	I^2^ = 86.5%; τ^2^ = 0.320
Maxillary first-molar distal tipping	4	5.33° (1.37–9.30)	I^2^ = 96.8%; τ^2^ = 5.974
Maxillary second-molar distal tipping	3	5.77° (−6.84–18.38)	I^2^ = 98.9%; τ^2^ = 25.464
Maxillary first-molar intrusion	3	0.58 mm (0.05–1.10)	I^2^ = 50.2%; τ^2^ = 0.023
Maxillary second-molar intrusion	2	0.67 mm (−1.14–2.48)	I^2^ = 25.4%; τ^2^ = 0.012
Mandibular first-molar distalization	3	1.87 mm (−0.32–4.06)	I^2^ = 79.7%; τ^2^ = 0.591
Mandibular second-molar distalization	2	1.96 mm (−2.06–5.97)	I^2^ = 37.9%; τ^2^ = 0.095

^1^ Values are presented as pooled single-group means estimated using random-effects models with corresponding 95% confidence intervals.

## Data Availability

No new data were created or analyzed in this study. Data sharing is not applicable to this article.

## Supplementary Materials

The following supporting information can be downloaded at https://www.mdpi.com/article/10.3390/dj14090611/s1, File S1, Complete Electronic Search Strategies; File S2, Standardized Data Extraction Items; Table S1, Studies Omitted During Full-Text Screening; Table S2, Characteristics of the Included Studies; Figure S1, Funnel plots for the primary meta-analyses; Table S3, Risk of bias summary; Table S4, GRADE Summary of Findings; Table S5, PRISMA 2020 checklist [44].

## Abbreviations

The following abbreviations are used in this manuscript:

CBCT	Cone-beam computed tomography
CI	Confidence interval
IPR	Interproximal reduction
OSF	Open Science Framework
REML	Restricted maximum likelihood
SD	Standard deviation
TAD	Temporary anchorage device
TMJ	Temporomandibular joint
TSAD	Temporary skeletal anchorage device
