# Supplementary material for: Clinical Outcomes of Sequential Distalization with Clear Aligners: A Systematic Review and Meta-Analysis
Source: Dent J (Basel). 2026 Sep 20;14(9):611. doi: 10.3390/dj14090611 (PMC13605400; doi:10.3390/dj14090611)
Supplement: Supplementary file 1 [file dentistry-14-00611-s001.zip › Figure S1.pdf]

Figure S1. Funnel Plots for the Primary Meta-Analyses

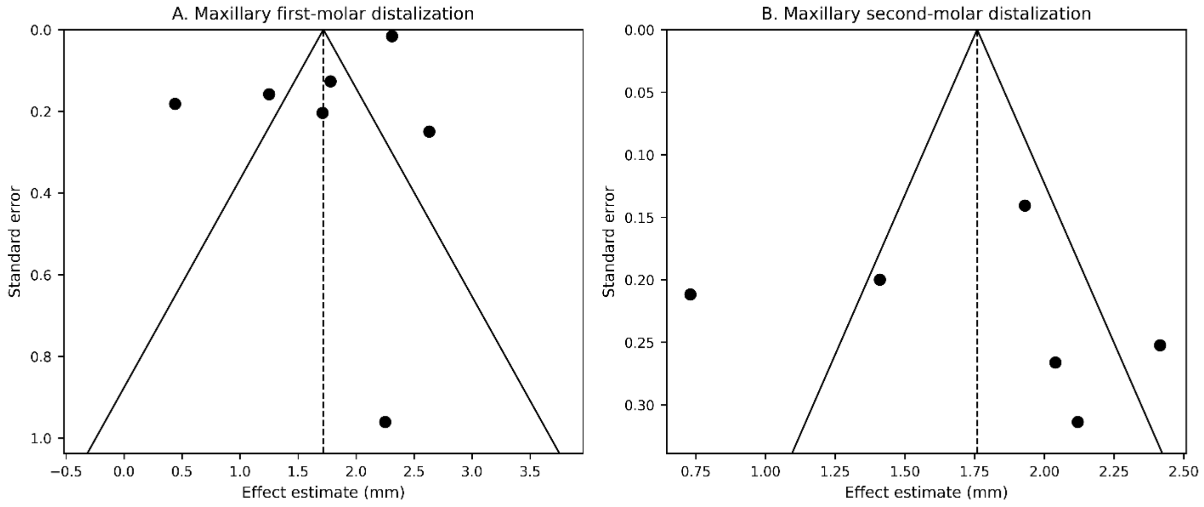

Note: Funnel plots for the primary meta-analyses of maxillary molar distalization. (A) Maxillary first-molar distalization; (B) maxillary second-molar distalization. The dashed vertical line represents the pooled effect estimate and the diagonal lines represent pseudo-95% confidence limits. Because fewer than 10 studies contributed to each analysis, visual assessment of funnel plot asymmetry should be interpreted cautiously.
