# Supplementary material for: Clinical Outcomes of Sequential Distalization with Clear Aligners: A Systematic Review and Meta-Analysis
Source: Dent J (Basel). 2026 Sep 20;14(9):611. doi: 10.3390/dj14090611 (PMC13605400; doi:10.3390/dj14090611)
Supplement: Supplementary file 1 [file dentistry-14-00611-s001.zip › File S1.pdf]

File S1: Complete Electronic Search Strategies

The electronic search strategies were developed to identify clinical studies evaluating sequential distalization with clear aligners. The searches were structured around two principal concepts: (1) clear aligner therapy and (2) orthodontic distalization. The initial strategy was developed in PubMed and refined through pilot searching to balance sensitivity and precision. The PubMed strategy was subsequently adapted for each database according to its indexing system, field codes, truncation conventions, and search syntax. Controlled vocabulary was incorporated when relevant indexed terms were available. Because clear aligner therapy and sequential distalization were not represented by sufficiently specific controlled vocabulary terms in all databases, controlled headings were combined with title, abstract, and keyword terms. The free-text component was retained to identify recently published and incompletely indexed records and to preserve specificity for clear aligners and distalization.

The following electronic databases were searched:

- PubMed
- Embase
- Scopus
- Web of Science Core Collection
- Cochrane Library

No restrictions were applied by publication date or language. The final searches were conducted on July 1, 2026. The reference lists of included studies and relevant systematic reviews were also examined to identify additional potentially eligible studies.

PubMed Search strategy

("Orthodontic Appliances, Removable"[Mesh] OR aligner*[Title/Abstract] OR "clear aligner"[Title/Abstract] OR "clear aligners"[Title/Abstract] OR Invisalign[Title/Abstract]) AND ("Tooth Movement Techniques"[Mesh] OR distaliz*[Title/Abstract] OR distalis*[Title/Abstract] OR "molar distalization"[Title/Abstract] OR "molar distalisation"[Title/Abstract] OR "maxillary molar distalization"[Title/Abstract] OR "mandibular molar distalization"[Title/Abstract] OR "sequential distalization"[Title/Abstract] OR "sequential distalisation"[Title/Abstract] OR "upper molar distalization"[Title/Abstract] OR "lower molar distalization"[Title/Abstract] OR "total distalization"[Title/Abstract] OR "arch distalization"[Title/Abstract])

PubMed search history format

#1 "Orthodontic Appliances, Removable"[Mesh]

#2 aligner*[Title/Abstract]

OR "clear aligner"[Title/Abstract]

OR "clear aligners"[Title/Abstract]

OR Invisalign[Title/Abstract]

#3 #1 OR #2

#4 "Tooth Movement Techniques"[Mesh]

#5 distaliz*[Title/Abstract]

OR distalis*[Title/Abstract]

OR "molar distalization"[Title/Abstract]

OR "molar distalisation"[Title/Abstract]

OR "maxillary molar distalization"[Title/Abstract]

OR "mandibular molar distalization"[Title/Abstract]

OR "sequential distalization"[Title/Abstract]

OR "sequential distalisation"[Title/Abstract]

OR "upper molar distalization"[Title/Abstract]

OR "lower molar distalization"[Title/Abstract]

OR "total distalization"[Title/Abstract]

OR "arch distalization"[Title/Abstract]

#6 #4 OR #5

#7 #3 AND #6

Embase Search strategy

In Embase, /exp instructs the database to search an Emtree term together with its narrower indexed terms. The :ti,ab,kw field codes restrict the free-text terms to titles, abstracts, and author keywords.

('removable orthodontic appliance'/exp OR aligner*:ti,ab,kw OR 'clear aligner':ti,ab,kw OR 'clear aligners':ti,ab,kw OR invisalign:ti,ab,kw) AND ('tooth movement'/exp OR distaliz*:ti,ab,kw OR distalis*:ti,ab,kw OR 'molar distalization':ti,ab,kw OR 'molar distalisation':ti,ab,kw OR 'maxillary molar distalization':ti,ab,kw OR 'mandibular molar distalization':ti,ab,kw OR 'sequential distalization':ti,ab,kw OR 'sequential distalisation':ti,ab,kw OR 'upper molar distalization':ti,ab,kw OR 'lower molar distalization':ti,ab,kw OR 'total distalization':ti,ab,kw OR 'arch distalization':ti,ab,kw)

Embase search history format

#1 'removable orthodontic appliance'/exp

#2 aligner*:ti,ab,kw

OR 'clear aligner':ti,ab,kw

OR 'clear aligners':ti,ab,kw

OR invisalign:ti,ab,kw

#3 #1 OR #2

#4 'tooth movement'/exp

#5 distaliz*:ti,ab,kw

OR distalis*:ti,ab,kw

OR 'molar distalization':ti,ab,kw

OR 'molar distalisation':ti,ab,kw

OR 'maxillary molar distalization':ti,ab,kw

OR 'mandibular molar distalization':ti,ab,kw

OR 'sequential distalization':ti,ab,kw

OR 'sequential distalisation':ti,ab,kw

OR 'upper molar distalization':ti,ab,kw

OR 'lower molar distalization':ti,ab,kw

OR 'total distalization':ti,ab,kw

OR 'arch distalization':ti,ab,kw

#6 #4 OR #5

#7 #3 AND #6

Scopus Search strategy

Scopus does not use a controlled-vocabulary thesaurus comparable to MeSH or Emtree. The strategy therefore searches titles, abstracts, and author keywords.

TITLE-ABS-KEY (aligner* OR "clear aligner" OR "clear aligners" OR Invisalign) AND TITLE-ABS-KEY (distaliz*OR distalis* OR "molar distalization" OR "molar distalisation" OR "maxillary molar distalization" OR "mandibular molar distalization" OR "sequential distalization" OR "sequential distalisation" OR "upper molar distalization" OR "lower molar distalization" OR "total distalization" OR "arch distalization")

Web of Science Core Collection Search Strategy

Web of Science does not use a controlled-vocabulary thesaurus. The Topic field searches titles, abstracts, author keywords, and Keywords Plus.

TS=(aligner* OR "clear aligner" OR "clear aligners" OR Invisalign) AND TS=(distaliz* OR distalis* OR "molar distalization" OR "molar distalisation" OR "maxillary molar distalization" OR "mandibular molar distalization" OR "sequential distalization" OR "sequential distalisation" OR "upper molar distalization" OR "lower molar distalization" OR "total distalization" OR "arch distalization")

Supplementary Search Methods

The reference lists of all included studies and relevant systematic reviews were examined to identify potentially eligible publications not retrieved through the electronic database searches. Additional records identified through citation searching were imported into Zotero, deduplicated, and transferred to Covidence for screening using the same eligibility criteria applied to records identified through the electronic database searches. The complete search strategies presented in this appendix reflect the final searches performed on July 1, 2026.
