# Supplementary material for: Clinical Outcomes of Sequential Distalization with Clear Aligners: A Systematic Review and Meta-Analysis
Source: Dent J (Basel). 2026 Sep 20;14(9):611. doi: 10.3390/dj14090611 (PMC13605400; doi:10.3390/dj14090611)
Supplement: Supplementary file 1 [file dentistry-14-00611-s001.zip › File S2.pdf]

File S2. Standardized Data Extraction Items

Domain	Variables Extracted
Study Identification	First author; year of publication; journal; country; funding source (if reported); conflicts of interest (if reported)
Study Characteristics	Study design; study setting; study period; sample size; number of treatment groups; duration of follow-up
Participant Characteristics	Mean age (or age range); sex distribution; malocclusion classification (e.g., Class II or Class III); inclusion and exclusion criteria; baseline orthodontic characteristics (if reported)
Intervention Characteristics	Clear aligner system (e.g., Invisalign, Spark, ClearCorrect, Angel Aligner); distalization protocol; arch treated (maxillary and/or mandibular); teeth distalized; sequential distalization sequence; attachments; interproximal reduction (IPR); use of Class II or Class III elastics; use of temporary anchorage devices (TADs); other adjunctive mechanics; treatment duration
Comparator Characteristics	Comparator intervention (if applicable); orthodontic appliance or treatment protocol; anchorage strategy
Primary Outcome	Amount of posterior tooth distalization
Secondary Outcomes	Anchorage preservation; movement predictability/accuracy; vertical changes; molar tipping; root resorption; periodontal outcomes; treatment duration; number of refinement aligners; occlusal outcomes; adverse events or complications; patient-reported outcomes (if available); post-treatment stability (if available)
Outcome Assessment	Measurement method (e.g., CBCT, lateral cephalometry, digital models, intraoral scanning); reference structures; time points evaluated
Methodological Quality	Risk-of-bias assessment; important study limitations identified by reviewers
Additional Notes	Author conclusions; reviewer comments; other relevant observations
