# Supplementary material for: Clinical Outcomes of Sequential Distalization with Clear Aligners: A Systematic Review and Meta-Analysis
Source: Dent J (Basel). 2026 Sep 20;14(9):611. doi: 10.3390/dj14090611 (PMC13605400; doi:10.3390/dj14090611)
Supplement: Supplementary file 1 [file dentistry-14-00611-s001.zip › Table S1.pdf]

Table S1. Studies Omitted During Full-Text Screening

**Author(s)**	**Reason**
Atalla et al. (2026)	Study type
Castroflorio et al., 2017	Not the outcome of interest
Chen et al., 2023	Insufficient extractable quantitative outcome data
Cheng et al. (2024)	Study type
Domini et al., 2025	Not the outcome of interest
Ebadi et al., 2025	Not the intervention of interest
Galant et al. (2025)	Study type
Gazzani et al., 2025	Not the intervention of interest
Jiang et al., 2021	Not the intervention of interest
Li et al., 2024	Not the intervention of interest
Lin et al., 2024	Not the intervention of interest
Lione et al., 2022	Not the intervention of interest
Liu et al., 2021	Not the intervention of interest
Marcelino et al., 2023	Not the intervention of interest
Meade et al., 2026	Not the intervention of interest
Nguyen, 2021	Not peer-reviewed
Shi et al. (2026)	Not the outcome of interest
Song et al. (2025)	Not the outcome of interest
Wang et al. (2025)	Not the outcome of interest
Yan et al. (2023)	Not the intervention of interest
Yi et al., 2018	Not the intervention of interest
