# Supplementary material for: Clinical Outcomes of Sequential Distalization with Clear Aligners: A Systematic Review and Meta-Analysis
Source: Dent J (Basel). 2026 Sep 20;14(9):611. doi: 10.3390/dj14090611 (PMC13605400; doi:10.3390/dj14090611)
Supplement: Supplementary file 1 [file dentistry-14-00611-s001.zip › Table S2.pdf]

Table S2. Characteristics of the Included Studies

Study	Country	Study design	Sample	Population	Intervention	Comparator	Assessment method	Primary outcome(s)
Al-Tayar et al. (2023)	China	Retrospective clinical study	23 adults (7 males, 16 females)	Skeletal Class I or II malocclusion with bilateral Class II molar relationship	Sequential maxillary molar distalization with Invisalign	None	CBCT (3D TMJ analysis)	Temporomandibular joint positional, dimensional, and volumetric changes after distalization
Al-Worafi et al. (2024a)	China	Retrospective clinical study	35 adults (140 maxillary molars)	Adult skeletal Class II malocclusion	Sequential maxillary molar distalization with Invisalign	None	CBCT	Alveolar bone thickness and orthodontically induced inflammatory root resorption
Al-Worafi et al. (2024b)	China	Retrospective comparative cohort study	70 adults (35 clear aligner; 35 fixed appliance)	Adult skeletal Class II malocclusion	Maxillary molar distalization with clear aligners	Fixed appliances with maxillary molar distalization	CBCT (3D TMJ analysis)	Comparison of TMJ positional and dimensional changes following clear aligner and fixed appliance treatment
Balboni et al. (2023)	Italy	Prospective clinical study	20 patients (13 females, 7 males)	Class II malocclusion	Sequential maxillary molar distalization with clear aligners	None	Lateral cephalometry	Vertical dentoskeletal and occlusal-plane effects
Caruso et al. (2019)	Italy	Retrospective clinical study	10 adults (8 females, 2 males)	Class II or end-to-end molar relationship	Sequential maxillary molar distalization with Invisalign	None	Lateral cephalometry	Vertical dentoskeletal effects and molar distalization

D'Antò et al. (2023)	Italy/Lithuania	Prospective clinical study	16 adults (4 males, 12 females)	Adult patients requiring maxillary molar distalization and derotation	Sequential maxillary molar distalization with Ordoline clear aligners	None	Digital intraoral models (STL), Geomagic Control X	Accuracy of maxillary first- and second-molar distalization and derotation
Elfouly et al. (2023)	Egypt/Canada	Retrospective clinical study	19 adults (61 maxillary molars)	Adult patients undergoing sequential maxillary molar distalization with Invisalign	Sequential maxillary molar distalization with Invisalign	None	CBCT	Relationship between maxillary sinus proximity and molar root resorption during distalization
Elfouly et al. (2024)	Egypt/Canada	Retrospective clinical study	19 adults (61 maxillary molars)	Adult patients undergoing sequential maxillary molar distalization with Invisalign	Sequential maxillary molar distalization with Invisalign	None	CBCT	Changes in molar angulation (tip), inclination (torque), and rotation following distalization
Hatami et al. (2026)	New Zealand	Retrospective clinical study	51 adults (subgroup of 12 for distalization analysis)	Adults with Class II subdivision malocclusion	Clear aligner treatment (Invisalign) for unilateral Class II correction	None	Digital models (ClinCheck + Geomagic Control X)	Accuracy of unilateral Class II correction, maxillary molar distalization, overjet, midline correction, and molar tipping

Keilig et al. (2024)	Germany	Retrospective non-interventional study	20 adults	Adults treated with sequential maxillary distalization using Invisalign	Sequential maxillary distalization with Invisalign	None	Digital models (ClinCheck + clinical casts) with palatal superimposition	Accuracy of planned distalization and associated vertical side effects
Lin et al. (2023)	Taiwan	Pilot retrospective clinical study	7 adults	Adults with mild-to-moderate Class II malocclusion treated non-extraction	Invisalign with sequential maxillary molar distalization and Class II elastics	None	Digital model-integrated CBCT with voxel-based superimposition	Accuracy of predicted versus achieved tooth movement, maxillary molar distalization, and skeletal/dental treatment changes
Lione et al. (2022)	Italy	Randomized prospective clinical trial	40 patients (20 clear aligners; 20 pendulum)	Patients with Class II malocclusion	Sequential maxillary molar distalization with clear aligners	Pendulum appliance	Lateral cephalograms	Comparison of vertical dentoskeletal changes during Class II treatment
Liu et al. (2024)	China	Retrospective comparative study	30 adults (17 miniscrews; 13 Class II elastics)	Adults with Class II malocclusion requiring whole maxillary arch distalization	Clear aligners with intramaxillary miniscrew anchorage	Clear aligners with Class II elastics	CBCT and digital dental models	Efficiency of whole maxillary arch distalization and comparison of miniscrew versus Class II elastic anchorage

Liu et al. (2025)	China	Preliminary retrospective clinical study	12 adults (19 U6 and 19 U7 evaluated)	Adults undergoing maxillary molar distalization with Invisalign	Sequential maxillary molar distalization with Invisalign and Class II elastics	None	iTero digital models with palatal superimposition	Amount of maxillary molar distalization, molar tipping, rotation, vertical changes, and anterior anchorage loss
Loberto et al. (2023)	Italy	Retrospective study	49 patients	Patients with Class II malocclusion undergoing maxillary molar distalization	Sequential maxillary molar distalization with clear aligners, attachments, and Class II elastics	None	3D digital casts (iTero) with palatal rugae registration	Premolar and canine anchorage loss during maxillary molar distalization
Maksyukov et al. (2026)	Russia	Randomized controlled clinical trial	40 adults (20 aligners + TADs; 20 aligners + Class II elastics)	Adults with Class II malocclusion requiring ≥2 mm maxillary molar distalization	Clear aligners with micro-implant anchorage	Clear aligners with Class II elastics	CBCT and 3D jaw model superimposition	Effectiveness of maxillary molar distalization, anchorage preservation, bodily versus tipping movement, and accuracy of digital treatment planning
Mamani et al. (2024)	Thailand	Preliminary retrospective study	14 adults	Adults treated with sequential maxillary molar distalization using Invisalign	Sequential maxillary molar distalization with clear aligners and Class II elastics	None	Lateral cephalometric superimposition and ClinCheck analysis	Planned versus achieved maxillary molar distalization and tipping (treatment accuracy)

Miao et al. (2023)	China	Prospective clinical study	22 adults (79 molars)	Adults undergoing sequential maxillary molar distalization	Clear aligners with sequential distalization, miniscrews, and Class I elastics	None	3D digital model superimposition using palatal rugae registration	Predicted versus achieved distal movement, intrusion, distal tipping, and crown buccal torque of maxillary molars
Qiang et al. (2025)	China	Retrospective study	28 adults	Adults undergoing bilateral maxillary molar distalization with clear aligners	Sequential maxillary molar distalization with clear aligners	Comparison of one-molar vs two-molar distalization staging and TADs vs Class II elastics vs no auxiliary anchorage	CBCT superimposition	Three-dimensional accuracy of crown and root displacement during maxillary molar distalization and influence of staging and anchorage strategies
Ravera et al. (2016)	Italy and Canada	Multicenter retrospective study	20 adults	Non-growing adults with bilateral end-to-end Class II molar relationship	Sequential maxillary molar distalization with Invisalign, attachments, and Class II elastics	None	Lateral cephalometric analysis	Maxillary molar distalization, molar tipping, vertical changes, skeletal effects, and incisor movement after treatment

Rongo et al. (2022)	Italy/Slovakia	Retrospective longitudinal clinical study	20 adults (15 females, 5 males; mean age 27.9 ± 7.5 years)	Adults with Class II malocclusion treated non-extraction	Invisalign with Class II intermaxillary elastics	None	Lateral cephalometry and digital dental models	Dentoalveolar, skeletal, and occlusal effects of Class II elastics during clear aligner treatment
Saif et al. (2026)	China	Prospective clinical study	30 adults (4 males, 26 females)	Non-growing adults requiring maxillary molar distalization followed by anterior alignment	Invisalign with sequential maxillary molar distalization and Class II elastics	None	Digital model superimposition (palatal rugae registration)	Accuracy of maxillary molar distalization, posterior anchorage loss, anterior tooth alignment, and molar stability
Shen et al. (2026)	United States/China	Retrospective clinical study	21 adults (17 females, 4 males; 38 distalization sites)	Adults requiring ≥1 mm programmed maxillary molar distalization	Clear aligners with infrazygomatic temporary skeletal anchorage devices (TSADs)	None	CBCT, intraoral scans, and digital model superimposition	Maxillary molar distalization efficacy, bodily movement, intrusion, tipping, rotation, and comparison of CBCT versus digital model measurements
Simon et al. (2014)	Germany	Retrospective split-mouth clinical study	30 patients (11 males, 19 females; mean age 32.9 years)	Patients requiring incisor torque, premolar derotation, or maxillary molar distalization with Invisalign	Invisalign with and without auxiliaries (attachments or Power Ridges)	Split-mouth comparison of auxiliaries versus no auxiliaries	Laser-scanned plaster models with three-dimensional surface superimposition	Accuracy of molar distalization, incisor torque, premolar derotation, and influence of attachments and staging

Wang et al. (2024)	China	Retrospective comparative cohort study	46 adults (22 clear aligners; 24 fixed appliances)	Adults with mild-to-moderate crowding undergoing miniscrew-assisted total arch distalization	Clear aligners with miniscrews	Fixed appliances with miniscrew-assisted total arch distalization	Lateral cephalometry	Comparison of dentoskeletal, dental, and soft-tissue changes following miniscrew-assisted molar distalization with clear aligners versus fixed appliances
Wu et al. (2021)	China	Retrospective clinical study	20 adults (8 males, 12 females)	Adults with mild-to-moderate mandibular crowding requiring non-extraction treatment	Mandibular molar distalization with Invisalign clear aligners (V-pattern sequential distalization)	None	CBCT with Dolphin 3D analysis	Effectiveness of mandibular first- and second-molar distalization, three-dimensional tooth movement, anterior anchorage changes, and facial soft- and hard-tissue changes
Yurdakul & Karslı (2024)	Türkiye	Prospective randomized clinical trial	24 adults (16 females, 8 males; 12 per group)	Adults with half-cusp Class II malocclusion	Sequential maxillary posterior distalization with clear aligners using 33% or 50% sequencing	33% sequencing vs 50% sequencing	Lateral cephalometry, digital models	Comparison of anterior anchorage loss, maxillary posterior distalization, distal tipping, transverse changes, and rotation using two sequential distalization protocols
Zhao et al. (2026)	China	Retrospective cross-sectional study	23 adults (15 females, 8 males)	Adults undergoing mandibular molar distalization with clear aligners	Mandibular molar distalization with Invisalign clear aligners (V-pattern sequential distalization)	None	CBCT with three-dimensional cephalometric analysis	Mandibular dentoskeletal changes, facial soft-tissue changes, mandibular molar distalization, and correlations with treatment duration and amount of distalization
