# Supplementary material for: Clinical Outcomes of Sequential Distalization with Clear Aligners: A Systematic Review and Meta-Analysis
Source: Dent J (Basel). 2026 Sep 20;14(9):611. doi: 10.3390/dj14090611 (PMC13605400; doi:10.3390/dj14090611)
Supplement: Supplementary file 1 [file dentistry-14-00611-s001.zip › Table S3.pdf]

Table S3. Risk of bias summary

Study	Study design	Risk-of-bias tool	Overall judgment or key appraisal findings
Al-Tayar et al. (2023)	Retrospective single-group	JBI	No concurrent control; no multiple pre/post measurements
Al-Worafi et al. (2024a)	Retrospective single-group	JBI	No concurrent control; no multiple pre/post measurements
Al-Worafi et al. (2024b)	Nonrandomized comparative	ROBINS-I	Serious
Balboni et al. (2023)	Prospective single-group	JBI	No concurrent control; no multiple pre/post measurements
Caruso et al. (2019)	Retrospective single-group	JBI	No concurrent control; no multiple pre/post measurements
D'Antò et al. (2023)	Prospective single-group	JBI	No concurrent control; no multiple pre/post measurements
Elfouly et al. (2023)	Retrospective single-group	JBI	No concurrent control; no multiple pre/post measurements
Elfouly et al. (2024)	Retrospective single-group CBCT study	JBI	No concurrent control; no multiple pre/post measurements
Hatami et al. (2026)	Retrospective single-group	JBI	No concurrent control; no multiple pre/post measurements; follow-up unclear
Keilig et al. (2024)	Retrospective single-group	JBI	No concurrent control; no multiple pre/post measurements
Lin et al. (2023)	Retrospective pilot study	JBI	No concurrent control
Lione et al. (2022)	Randomized controlled trial	RoB 2	Some concerns
Liu et al. (2024)	Nonrandomized comparative	ROBINS-I	Serious
Liu et al. (2025)	Single-group clinical study	JBI	No concurrent control
Loberto et al. (2023)	Retrospective single-group	JBI	No concurrent control; no multiple pre/post measurements
Maksyukov et al. (2026)	Randomized controlled trial	RoB 2	Some concerns
Mamani et al. (2024)	Retrospective single-group	JBI	No concurrent control; no multiple pre/post measurements
Miao et al. (2023)	Retrospective single-group	JBI	No concurrent control; no multiple pre/post measurements
Qiang et al. (2025)	Retrospective single-group	JBI	No concurrent control; no multiple pre/post measurements
Ravera et al. (2016)	Retrospective single-group	JBI	No concurrent control; no multiple pre/post measurements
Rongo et al. (2022)	Retrospective single-group	JBI	No concurrent control; no multiple pre/post measurements
Saif et al. (2026)	Prospective single-group	JBI	No concurrent control
Shen et al. (2026)	Prospective single-group	JBI	No concurrent control; no multiple pre/post measurements
Simon et al. (2014)	Split-mouth clinical study	JBI	No concurrent control; no multiple pre/post measurements; follow-up not complete for all evaluated movements
Wang et al. (2024)	Nonrandomized comparative	ROBINS-I	Serious

Wu et al. (2021)	Retrospective single-group	JBI	No concurrent control; no multiple pre/post measurements
Yurdakul & Karslı (2024)	Randomized controlled trial	RoB 2	Some concerns
Zhao et al. (2026)	Retrospective single-group	JBI	No concurrent control; no multiple pre/post measurements

Note: RoB 2, Cochrane Risk of Bias 2; ROBINS-I, Risk Of Bias In Non-randomized Studies of Interventions; JBI, Joanna Briggs Institute Critical Appraisal Checklist for Quasi-Experimental Studies; CBCT, cone-beam computed tomography. For studies assessed using the JBI checklist, item-level findings are reported rather than an overall categorical judgment because the checklist does not specify thresholds for deriving global ratings.
