# Supplementary material for: Clinical Outcomes of Sequential Distalization with Clear Aligners: A Systematic Review and Meta-Analysis
Source: Dent J (Basel). 2026 Sep 20;14(9):611. doi: 10.3390/dj14090611 (PMC13605400; doi:10.3390/dj14090611)
Supplement: Supplementary file 1 [file dentistry-14-00611-s001.zip › Table S4.pdf]

Table S4. GRADE Summary of Findings

Outcome	Evidence	Risk of bias	Inconsistency	Indirectness	Imprecision	Publication bias	Certainty
Maxillary first-molar distalization	7 studies; pooled 1.72 mm (95% CI, 1.02–2.42)	Serious	Very serious	Not serious	Not serious	Not assessed	Very low ⨁◯◯◯
Maxillary second-molar distalization	6 studies; pooled 1.76 mm (95% CI, 1.12–2.40)	Serious	Serious	Not serious	Not serious	Not assessed	Very low ⨁◯◯◯
Maxillary molar distal tipping	Exploratory pooled analyses; U6: 5.33° (95% CI, 1.37°–9.30°); U7: 5.77° (95% CI, −6.84° to 18.38°)	Serious	Very serious	Not serious	Serious	Not assessed	Very low ⨁◯◯◯
Maxillary molar intrusion	Limited evidence; U6: 0.58 mm (95% CI, 0.05–1.10); U7: 0.67 mm (95% CI, −1.14 to 2.48)	Serious	Serious	Not serious	Serious	Not assessed	Very low ⨁◯◯◯
Mandibular molar distalization	3 studies; L6: 1.87 mm (95% CI, −0.32 to 4.06); L7: 1.96 mm (95% CI, −2.06 to 5.97)	Serious	Serious	Serious	Very serious	Not assessed	Very low ⨁◯◯◯
Movement predictability/accuracy	Multiple studies; predictability was reported using percentage accuracy, planned-versus-achieved displacement, movement expression, derotation, and prediction error, with differing assessment time points.	Serious	Very serious	Serious	Serious	Not assessed	Very low ⨁◯◯◯
Anchorage preservation/loss	Randomized and observational evidence; outcomes included incisor proclination and protrusion, overjet changes, canine mesialization, and posterior anchorage loss, with differing distalization and anchorage protocols.	Serious	Serious	Serious	Serious	Not assessed	Very low ⨁◯◯◯
Biological effects/safety	Limited evidence evaluating root resorption, alveolar bone changes, and TMJ outcomes, with different biological outcomes assessed across studies.	Serious	Serious	Serious	Very serious	Not assessed	Very low ⨁◯◯◯
Long-term stability	Very limited longitudinal evidence; post-distalization changes were evaluated in few studies, with limited long-term follow-up.	Serious	Serious	Serious	Very serious	Not assessed	Very low ⨁◯◯◯

Abbreviations: CI, confidence interval; GRADE, Grading of Recommendations Assessment, Development and Evaluation; L6, mandibular first molar; L7, mandibular second molar; TMJ, temporomandibular joint; U6, maxillary first molar; U7, maxillary second molar.

Notes: Certainty of evidence was assessed across the GRADE domains of risk of bias, inconsistency, indirectness, imprecision, and publication bias. Publication bias was not formally assessed because fewer than 10 studies contributed to each outcome. Very-low-certainty evidence indicates very limited confidence in the estimated effect; the true effect may be substantially different from the estimate. For maxillary second-molar tipping, imprecision was additionally reflected by the wide 95% CI (−6.84° to 18.38°).
